# Characterization of Gas Absorption Modules Based on Flexible Mid-Infrared Hollow Waveguides

**DOI:** 10.3390/s19071698

**Published:** 2019-04-10

**Authors:** Kewang Chen, Zeqiao Zhao, Xuewen Zhang, Xian Zhang, Xiaosong Zhu, Yiwei Shi

**Affiliations:** Key Laboratory for Information Science of Electromagnetic Waves (MoE), Fudan University, Shanghai 200433, China; 16213010010@fudan.edu.cn (K.C.); 18210720082@fudan.edu.cn (Z.Z.); 17210720180@fudan.edu.cn (X.Z.); 18110720057@fudan.edu.cn (X.Z.); zhuxiaosong@fudan.edu.cn (X.Z.)

**Keywords:** substrate-embedded hollow waveguide, spectroscopic gas sensing, modularization, mid-infrared

## Abstract

A new gas absorption module, the substrate-embedded hollow waveguide (eHWG) model, is proposed. It consists of a substrate with a curved channel and a hollow waveguide. The hollow waveguide is curved into the channel and works as a gas absorption cell as well as a transmission medium for mid-infrared light. Owing to the low loss property of the hollow waveguide, the signal-to-noise ratio (SNR) was improved for the sensing system. A polycarbonate (PC) base tube was used to obtain flexibility in the fabrication of the hollow waveguide. A silver (Ag) layer and a silver iodide (AgI) layer were inner-coated to ensure a low loss property at the fingerprint wavelength of methane gas. A sensing system was established using a Fourier transform infrared spectrometer (FTIR), an external detector, and an eHWG. Experimental investigations were carried on the sensing performance of eHWGs with various channel shapes. Comparison studies were made on eHWGs embedded with Ag-coated or Ag- and AgI-coated hollow waveguides. The Ag- and AgI-coated hollow waveguides with inner diameters of 0.7, 1.4, and 2.0 mm were used in the eHWGs. The large bore waveguide had low loss but high bending additional loss. The large bore waveguide had a low detection limit due to high coupling efficiency with the light source. A limit of detection (LOD) as low as 2.7 ppm was attained for the system using the eHWG with the long and large bore waveguide.

## 1. Introduction

Spectroscopic gas sensing in the mid-infrared (MIR) regions is an attractive gas detection method due to its intrinsic molecular selectivity, high sensitivity, fast response, and non-toxic characteristics [1,2]. The Beer–Lambert law describes the relationship between light absorbance and gas concentration [3,4]. The absorption cell is one of the most important parts in a spectroscopic sensing system. Compared to traditional absorption cells—such as the Herriott cavity, White cavity, Chernin cavity, and integrating sphere—waveguide cells have drawn greater attention in recent years [5,6] due to their low loss, flexibility, and small inner diameter. Furthermore, dielectric-coated metallic hollow waveguides [7,8] have a low loss property in the MIR regions, in which many hazardous gases have strong absorptions. It has been shown that dielectric-coated metallic waveguides attain lower loss than metallic waveguides. The loss is normally 0.1–1 dB/m for various mid-infrared lasers in the wavelength of 1.0–11 μm, compared to the loss of more than 3 dB/m for a metallic waveguide. Flexibility makes it possible to coil a long waveguide into a small space to realize a miniaturized gas absorption cell.

The substrate-integrated hollow waveguide (iHWG) is a recently proposed gas absorption cell [9]. An iHWG is a gas absorption module with a fixed size and standardized input/output coupling. In terms of the sensing principle, it is a curved metallic waveguide for simultaneous infrared light transmission and gas absorption. A series of articles have been published on the performance and optimization of iHWG [10,11,12,13,14,15]. It shows great potential for the application as a gas absorption cell with flexibility and modularity. Standardization for the iHWG is expected in the industrial and medical fields, where it is a standard part in spectroscopic sensing systems. It allows very convenient substitution in a sensing system when aiming to detect various gases and liquids at their corresponding fingerprint wavelengths.

Theoretical and experimental studies have shown [16,17] that dielectric-coated metallic hollow waveguides have much smaller transmission loss than metallic hollow waveguides, especially when coiled into small circles. Transmission loss of the absorption cell is important to the system. Low loss means a high signal-to-noise ratio (SNR) and leads to a low limit of detection (LOD).

In this study, a new gas absorption module was proposed, namely a substrate-embedded hollow waveguide (eHWG). A polycarbonate base tube was chosen to achieve flexibility for the big-bore hollow waveguide. Four kinds of substrates with various channel shapes were designed and fabricated referring to the iHWG [18]. The dielectric-coated metallic waveguide was used to substitute the metallic channel in the iHWG. High SNR and low LOD were expected compared to the iHWG due to the low loss property of the waveguide in the mid-IR region.

## 2. Fabrication

### 2.1. Hollow Waveguide

A metallic hollow waveguide, such as the curved channel in an iHWG, was one of the choices for the absorption cell. A dielectric-coated metallic hollow waveguide would be a better choice because of ist higher transmittance. Research has shown [16,17] that the latter not only has lower loss, but also lower bending additional loss than the former. For a dielectric-coated metallic hollow waveguide, silver is commonly used as the metallic material. There are several choices for the dielectric layer, including non-organic materials such as AgI, CuI, ZnS, and ZnSe, and organic materials such as cyclic olefin polymer (COP), polystyrene (PS), polyimide (PI), fluorocarbon polymer (FCP), and ethylene-vinyl acetate (EVA). Among these kinds of hollow waveguide, the AgI/Ag waveguide is one of the best choices due to its easy fabrication and small material absorption in a wide wavelength region from near-IR to mid-IR.

Fabrication techniques for an AgI/Ag waveguide can be found in the literature [19,20,21,22]. A silver layer is firstly plated on the inner wall of a base capillary tube using the silver mirror reaction. Then, an iodization process is adopted to turn the upper part of the silver layer into silver iodide layer. The thickness of the Ag layer in the waveguide was around 250 nm, much thicker than the skin depth. The AgI film thickness is one of the key parameters to ensuring attaining low loss at a target wavelength. Theoretically, the optimum film thickness *d* of the AgI film for an operation wavelength *λ*_0_ is [16]

(1) $$d=\frac{\lambda_{0}}{2\pi \sqrt{n_{}^{2}-1}}\tan^{-1}\left[\frac{n}{{\left(n_{}^{2}-1\right)}^{1/4}}\right]$$

where *n* is the refractive index of AgI at the wavelength *λ*_0_.

In the fabrication process, the thickness of the AgI film was controlled by the fabrication parameters, such as the concentration of the iodine solution, reaction temperature, and reaction time. In this paper, methane was the target gas, with fingerprint absorption at the wavelength of 3.31 μm. The optimum AgI film thickness was 0.29 μm.

### 2.2. Module Substrates

**Figure 1:**
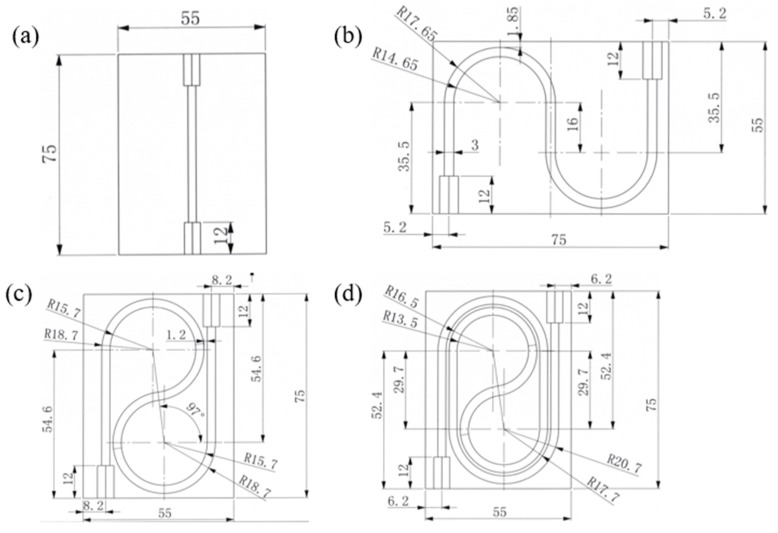
Detailed design of the module substrates: (**a**) straight, (**b**) two semicircle, (**c**) one circle, and (**d**) two circles. (Unit: mm).

Referring to the iHWGs, four kinds of module substrates were designed, as shown in Figure 1. The four modules had different channel shapes. They were chosen by considering the factors of channel length and bending configuration to compare the sensing performance. 

The substrates were aluminum plates of the same size: 75 × 55 × 9 mm (L × W × H). Channels on the substrate was made by precise machining and drilling. The channel lengths for the substrates of (a) to (d) were 7.5 cm, 18.5 cm, 26.5 cm, and 42 cm, respectively. The long channel has long optical length and high sensitivity. However, the long channel has to be sharply bent in the small substrate. The sharp bending brings additional transmission loss, which leads to a small SNR ratio, and thus low LOD. There is a trade-off for the situation and this trade-off changes when directed at gases with different concentrations.

### 2.3. eHWG Series

There are two methods to fabricate the eHWG. One is to fabricate a glass base tube with a fixed shape that is the same as the substrate channel. The glass base tube is simply placed in the channel. Then the inner layers of Ag and AgI are coated. The other method is to fabricate an extremely flexible AgI/Ag waveguide and curve the waveguide into the channel. The first method is simple but causes difficulties in the inner coating processes in a sharply curved tube. The second method is challenging in terms of the waveguide fabrication technique, because a large bore size and flexibility are required for the waveguide. Although the small bore waveguide is flexible, it causes large transmission losses and difficulty in coupling with a light source.

**Figure 2:**
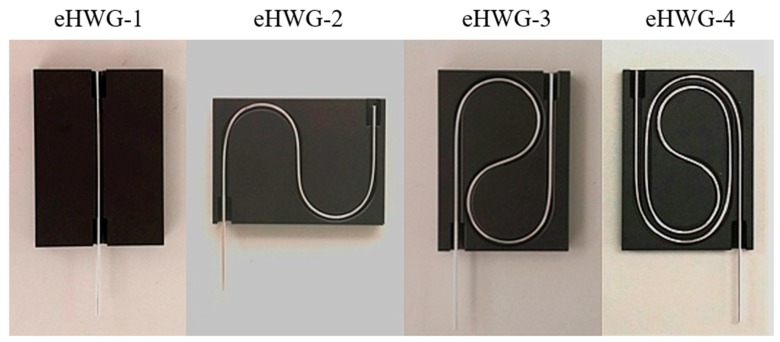
eHWG (no. 1 to 4) with flexible waveguide.

Polycarbonate (PC) was selected as the base tube material because it is more flexible than a glass capillary, and there is no debris even when a PC tube breaks due to sharp bending. The inner diameter (ID), outer diameter (OD), and wall thickness were carefully selected to achieve flexibility and robustness [23]. Flexible Ag and AgI/Ag waveguides were fabricated and curved into the substrates, as shown in Figure 2.

The length of the waveguide in the eHWG-1 to −4 were 10.5 cm, 21.5 cm, 29.5 cm, and 45 cm, respectively. We used hollow waveguides with 3 cm extra length that stretched out of the substrate to make it easy to couple the output IR light into the deuterated triglycine sulfate (DTGS) detector. It can be seen in Figure 2 that the extreme flexibility of the hollow waveguides made it possible to curve the waveguide into the sharply bent channel. The minimum bending radius was as small as 1.5 cm in eHWG-4. Furthermore, the channel in the substrate was used only for supporting the waveguide. Therefore, there was no need for precision machining and polishing of the channel surface. The substrate was able to be less expensively fabricated using the simple method of thermoplastic molding.

## 3. Measurement

**Figure 3:**
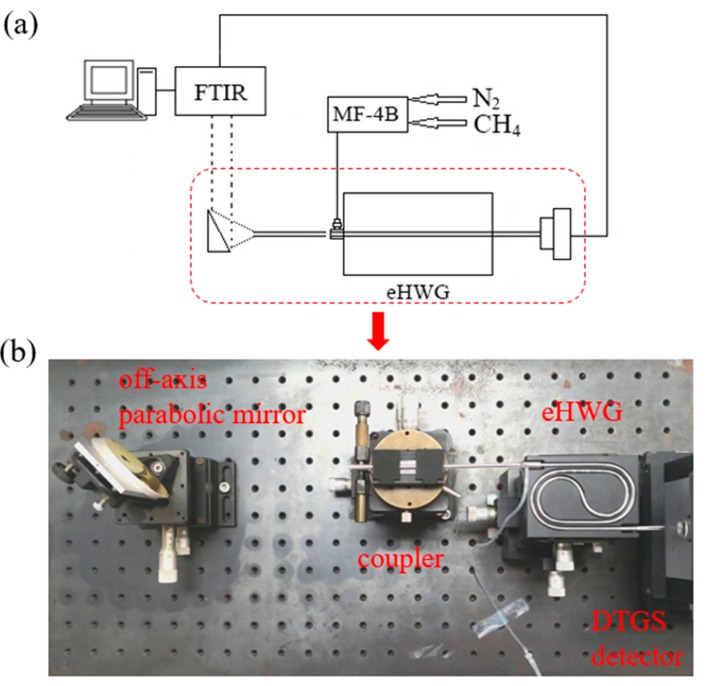
(**a**) Diagram of the sensing system. (**b**) Picture of light coupling, gas let in at the coupling end, gas let out at the distal end, and the detector for the eHWG.

A sensing system was established as shown in Figure 3, using eHWG as a gas absorption cell. A Fourier transform infrared spectrometer (FTIR) system, model Bruker Vertex 70, was used as the light source and for output detection. An external DTGS detector was used to measure the output from the eHWG. We can see in Figure 3 that the extra 3 cm long waveguide was retained and stretched out of the substrate for easy and repeatable coupling with the DTGS detector. Methane (CH_4_) gases with various concentrations were obtained using a 3000 ppm methane gas and a dilution nitrogen gas. A gas dilution instrument (a national standard device GB MF-4B) was used for producing methane gases with various concentrations.

The measuring processes were as follows: (1) Alignment. A 12 cm-long Ag waveguide was used as the coupler. The coupler had the same ID as that of the embedded waveguide in the eHWG. The IR light was coupled into the eHWG through a KCl window sealing. Alignment was carefully carried out for the coupler and the eHWG to achieve the best coupling and maximum output. (2) The system background was measured after a nitrogen gas purge through the waveguide cell for 60 seconds. (3) Methane gas generated from the gas dilution instrument was let into the eHWG. The absorption intensity was measured for gases with various concentrations. The flow rate for the measured gas was 1900 ml/min.

### 3.1. Loss Property

**Figure 4:**
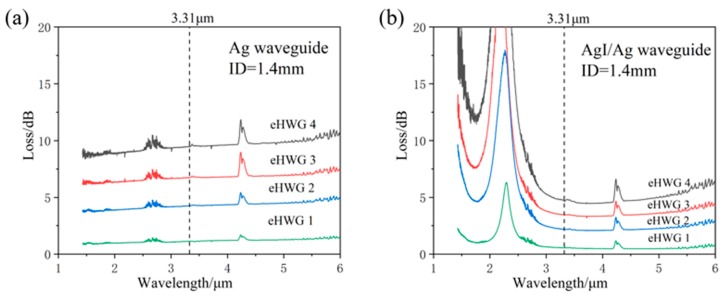
Loss spectra of the eHWGs with (**a**) an Ag waveguide or (**b**) an AgI/Ag waveguide.

**Figure 5:**
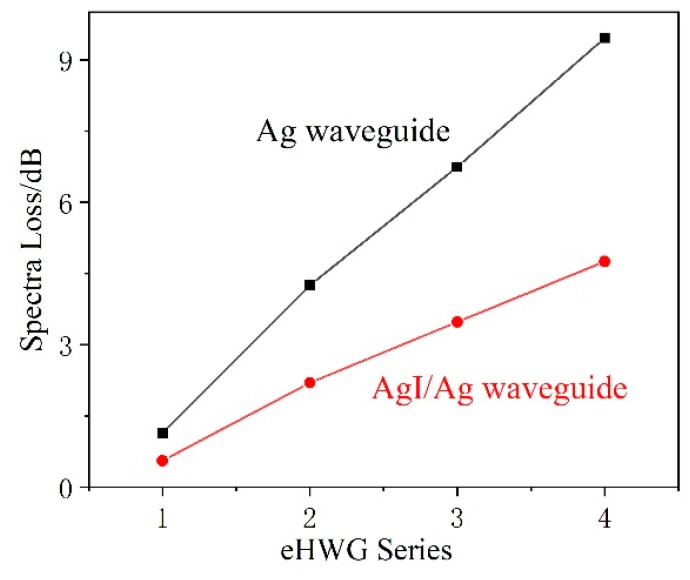
Spectra loss of the eHWGs at the wavelength of 3.31 μm.

The Ag and AgI/Ag waveguides were fabricated and were curved into the substrates. Figure 4a,b present the measured loss spectra of the Ag and AgI/Ag waveguides in four kinds of eHWGs. It can be seen that the eHWG with the Ag waveguide had a flat loss property in the near and mid-IR regions. The eHWG with the AgI/Ag waveguide had an interference peak due to the transparent AgI film on the Ag layer. The small peaks at 4.2 μm were due to the absorption of CO_2_ gas in the air. The impact of this can be ignored in the context of this research. It can also be seen that the longer waveguide or sharper bending waveguide had higher loss. Figure 5 shows the spectra losses for the eHWGs. It is obvious that the eHWG with the Ag waveguides had higher loss than the eHWG with AgI/Ag waveguides. Furthermore, compared to the rapid loss increase of the Ag waveguides in eHWG-1 to -4, a slow growth of loss can be seen for the eHWG with the AgI/Ag waveguides. This is the merit of using AgI/Ag waveguides over Ag waveguides. 

**Figure 6:**
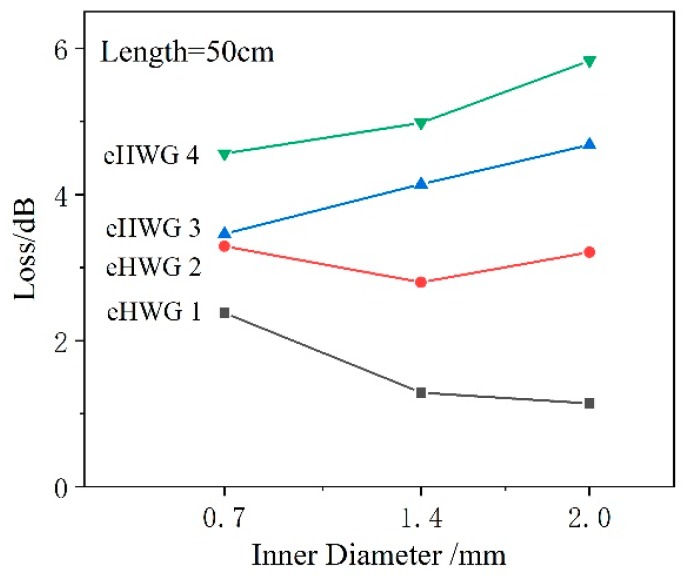
Spectra losses of eHWGs with a waveguide with various IDs.

Another measured result concerned the influence of the ID of the waveguide. Three kinds of AgI/Ag waveguides were fabricated. They had the same length of 50 cm, but different IDs of 0.7 mm, 1.4 mm, and 2.0 mm, respectively. The middle part of the waveguides was curved into the substrate channel (eHWG-1 was straight, without a curve). Table 1 provides a summary of the spectra losses at the wavelength 3.31 μm. Figure 6 shows the loss as a function of ID for the four eHWGs. eHWG-1 showed loss decreases with the increase in the ID, because the large bore waveguide had low loss. eHWG-2 showed little loss variation because the ID increase offset the additional loss caused by the bending. For eHWG-3 and eHWG-4, which had sharply curved channels, the loss increased with the bore size because the ID increase was not sufficient to offset the additional loss caused by bending.

### 3.2. Absorbance 

**Figure 7:**
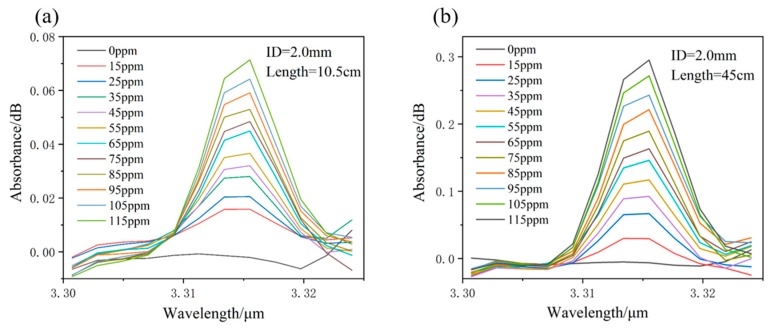
Absorption peaks for methane gases of different concentrations. (**a**) eHWG-1; (**b**) eHWG-4 with AgI/Ag waveguides.

The background of the system was firstly measured, as shown in Figure 3, after a nitrogen gas purge in the waveguide. Then, methane gas with various concentrations flowed into the waveguide cell. The absorption was measured and the typical measured results for eHWG-1 and -4 are shown in Figure 7. The absorbance was recorded as the peak value of the absorption in the wavelength range of 3.305–3.322 μm. The absorbance became stronger with the increase in the waveguide length, as well as with the increase in the methane gas concentration.

At first, eight groups of data were measured for eHWG-4 with Ag or AgI/Ag waveguides with an ID of 1.4 mm. The data were used for a comparison analysis of the performance of the eHWG with Ag or AgI/Ag waveguides. Then 12 groups of data (including four groups of data in the abovementioned measurements) were measured for four eHWGs with AgI/Ag waveguides with IDs of 0.7 mm, 1.4 mm, and 2.0 mm.

## 4. Discussion

### 4.1. eHWG with Ag or AgI/Ag Waveguides

**Figure 8:**
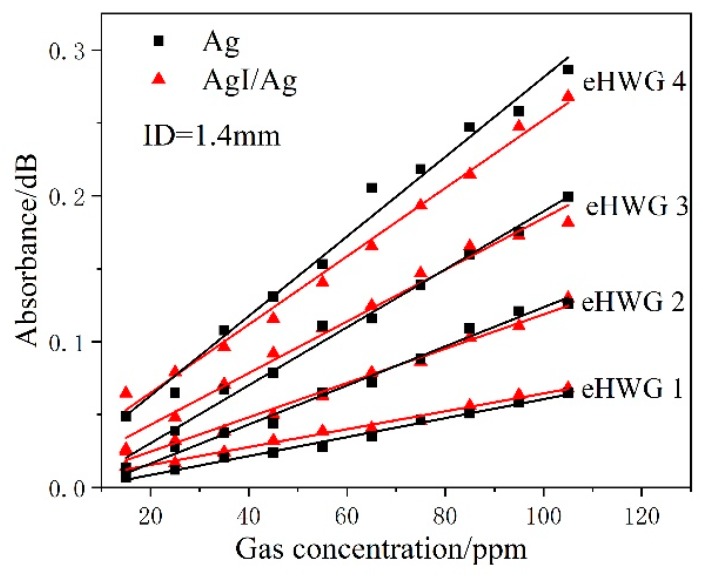
Absorbance vs. gas concentration for the eHWGs with an Ag or AgI/Ag waveguide.

Eight groups of data were measured using four eHWGs with Ag or AgI/Ag waveguides with an ID of 1.4 mm. Figure 8 shows the absorbance versus concentration. eHWG-4 showed the fastest absorbance increase in concentration compared to eHWGs-1, -2, and -3, because eHWG-4 had the longest waveguide cell. For the same eHWG with an Ag or AgI/Ag waveguide, a similar absorbance increase was observed. This indicates that they had the same sensitivity due to their having the same waveguide length. We note that the eHWG with an Ag waveguide had a slightly higher sensitivity (larger slope) compared to the AgI/Ag waveguide. This might be caused by the multimode delivery in the waveguide. More modes were excited in the Ag waveguide than in the AgI/Ag waveguide. The effective optical length [24] of the Ag waveguide was a little longer than that of the AgI/Ag waveguide. However, the eHWG with an AgI/Ag waveguide had a lower LOD due to its low loss, as we will see in the following discussion.

Table 2 provides a summary of the performance of the eHWGs with an Ag or AgI/Ag waveguide. The standard deviation of the blank signal (SD of blank) and the LOD of the system was calculated using the same method for the iHWG [9,10]. The method supports the eHWG because uses the same mechanism as the iHWG in the detection of the gas concentration. 

The SD of blank was calculated based on the system background [9,10]. The data were taken from the system background in the methane absorption wavelength region of 3.305–3.322 μm when the methane concentration was 0 ppm. When the noise was zero, the system background of 0 ppm was a straight line of zero. However, the noise in the background caused fluctuations in the loss spectrum. Thus it was necessary to calculate the SD of blank at this time to estimate the LOD. Sensitivity is the slope of the fitting line for the absorbance versus concentration. The LOD was calculated using Equation (2) according to the IUPAC method [25]. When the confidence factor was 3, the allowable confidence level was 99.86%.

(2) $$\mathrm{LOD}=\frac{3\;\times \;\left(\mathrm{SD}\;\mathrm{of}\;\mathrm{blank}\right)}{\mathrm{Sensitivity}}$$

**Figure 9:**
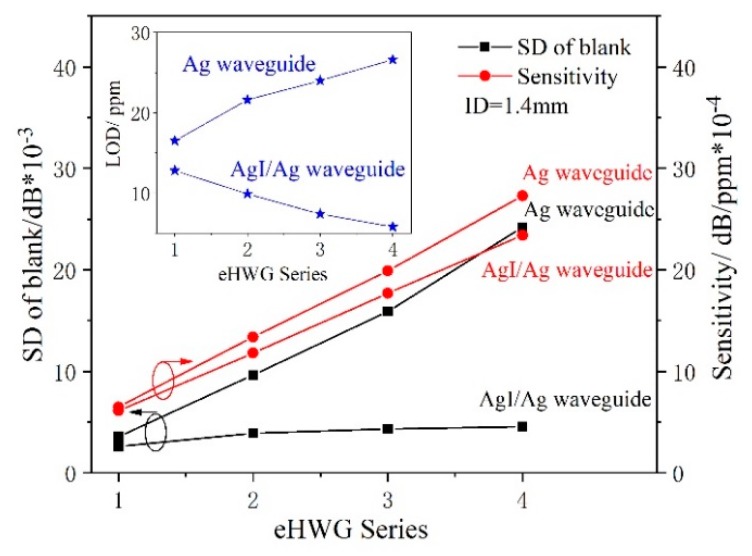
Sensitivity and LOD of eHWG with an Ag or AgI/Ag waveguide.

Figure 9 shows several important results according to the data in Table 2. It can be seen that the sensitivity was almost the same for the same eHWG regardless of whether it had an Ag or AgI/Ag waveguide (red lines). A rapid increase in the SD of blank was observed from eHWG-1 to -4 when they were embedded with Ag waveguides. The increase in the SD of blank was much slower when embedded with AgI/Ag waveguides (black lines). Consequently, the LOD decreased from eHWG-1 to -4 with the AgI/Ag waveguide because of longer waveguide cell, as shown in the inset in Figure 9. On the other hand, the LOD increased from eHWG-1 to -4 with Ag waveguides because the high loss caused low SNR and a large SD of blank, which overwhelmed the sensitivity increase of the long waveguide cell. The same result is found in the literature [13] for iHWG with a metallic channel. Based on the results, the AgI/Ag waveguide was a reasonable choice in order to seek to ensure a low LOD.

### 4.2. eHWG with AgI/Ag Waveguides with Various IDs 

**Figure 10:**
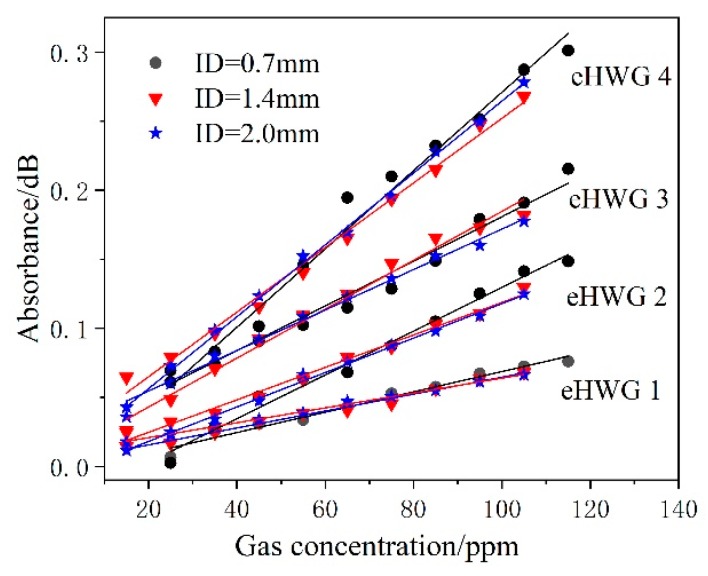
Absorbance vs. gas concentration for the eHWGs with AgI/Ag waveguides with different IDs.

It was shown above that choosing the AgI/Ag waveguide has advantages over the Ag waveguide at the LOD. The performance of the eHWG with AgI/Ag waveguides of various IDs were also experimentally discussed. Twelve groups of data (including four groups of data in Figure 8) were measured for four eHWGs with AgI/Ag waveguides with IDs of 0.7 mm, 1.4 mm, and 2.0 mm. The absorbance versus concentration is shown in Figure 10. Similar results were obtained, showing that the eHWGs with longer waveguides had higher sensitivity. Concerning the sensitivity of the same eHWG with waveguides with different IDs, we concluded that the inner diameter had no obvious influence on the sensitivity. Although a slight difference in sensitivity can be observed in Figure 10 for waveguides with various IDs in the same eHWG, this might have been caused by measuring error, or the different mode structures and coupling conditions for the waveguides.

**Figure 11:**
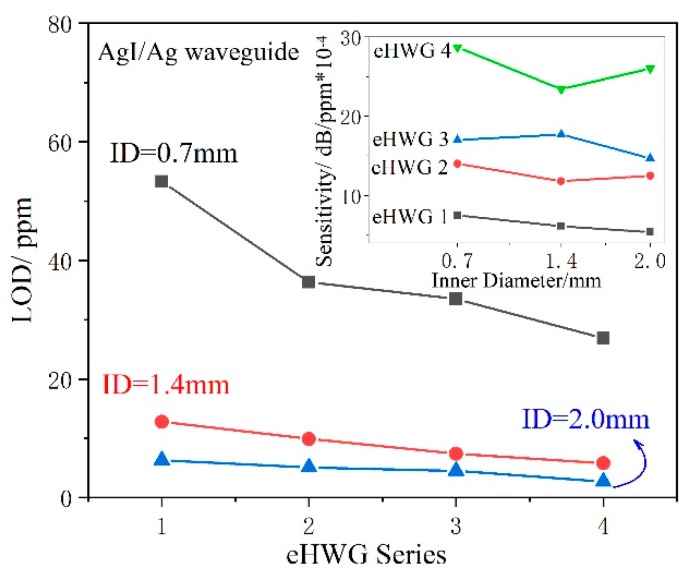
Characteristics of the eHWGs with AgI/Ag waveguides with different IDs.

Table 3 presents a summary of the performance of the eHWGs with AgI/Ag waveguides with various IDs. Figure 11 presents several important results drawn from the data in Table 3. It can be seen in the inset of Figure 11 that the sensitivity increased from eHWG-1 to −4 due to the long waveguide, while no obvious influence of the ID on sensitivity can be seen for the same eHWG.

Figure 6 shows that the transmission loss of the eHWG increased as the degree of bending increased, especially as the loss of the large bore waveguide increased rapidly. However, the large bore waveguide had high coupling efficiency, the signal-to-noise ratio of the system was increased, and the SD of the blank became small. Therefore, the eHWG with a large bore waveguide had a small LOD. We also note that there was a large LOD decrease when the ID changed from 0.7 mm to 1.4 mm, but the decrease was much smaller when the ID changed from 1.4 mm to 2.0 mm. This was because large bore waveguide suffered large additional bending loss. Normally, a long waveguide has high sensitivity and a large bore has a low SD of blank, but a long waveguide has to be bent sharply in a small space and a large bore waveguide lacks flexibility.

The LOD relates to the sensitivity and the SD of blank. ID, flexibility, and length need to be considered comprehensively for a certain eHWG. It is beneficial to practical applications that the large bore waveguide is easy to couple with inexpensive IR light sources and detectors.

## 5. Conclusions

Extremely flexible hollow waveguides were fabricated that curve into a small substrate. They formed a substrate-embedded hollow waveguide (eHWG) as a miniaturized gas absorption module. The waveguides were inner-coated with Ag and AgI optical film to obtain minimum transmission loss at the fingerprint wavelength of 3.31 μm for methane gas detection. An experimental sensing system was established using an FTIR system, DTGS detector, and the eHWG. Four kinds of eHWGs with flexible Ag and AgI/Ag waveguides with various IDs were used as the gas absorption cell. The performance of the system using different eHWGs was analyzed.

It was shown that the eHWGs with Ag and AgI/Ag waveguides had a similar sensitivity because the waveguides had the same length. The eHWGs with AgI/Ag waveguides had lower LODs because of the low loss and high SNR. Compared to the Ag waveguide, the AgI/Ag waveguide had both small straight loss and bending additional loss. Waveguides with a larger ID had lower straight loss but higher bending additional loss. Therefore, waveguides with larger IDs may have had a larger loss than small-bore waveguides when curved into a sharply bent substrate channel. There is also a limit for the ID, because large bore waveguides tend to be rigid and lack flexibility. In this research, eHWG 4 had the longest waveguide and the sharpest bending. The LOD of the system using eHWG-4 was 2.7 ppm. This was one of the best results found for the sensing system using the FTIR and DTGS detector, compared to 6 ppm using iHWG [10]. 

An eHWG with a AgI/Ag waveguide is a simple but convenient gas cell. The standardized size and input/output interface provide great convenience in many applications. The low cost of plastic thermoforming for the substrate fabrication and easy substitution of the embedded waveguide provide flexibility for the detection of different gases. The new module has several advantages over other absorption cells in the field of spectroscopic gas sensing, such as its small gas volume, low LOD, low cost, and stable coupling with the source and detector in engineering. It is also convenient to change the embedded AgI/Ag waveguide for different wavelength transmission and sensing.

## Figures and Tables

**Table 1:** Spectra losses of the waveguide with different IDs (length = 50 cm).

ID (mm)	eHWG-1 (dB)	eHWG-2 (dB)	eHWG-3 (dB)	eHWG-4 (dB)
0.7	2.38	3.29	3.46	4.56
1.4	1.29	2.80	4.14	4.98
2.0	1.14	3.21	4.68	4.83

**Table 2:** Performances for eHWG with an Ag or AgI/Ag waveguide (ID = 1.4 mm).

Waveguide Type	eHWG series	SD of Blank (dB*10^−3^)	Sensitivity (dB/ppm*10^−4^)	LOD (ppm)	Correlation Coefficient
Ag	eHWG-1	3.562	6.5	16.5	0.98894
Ag	eHWG-2	9.644	13.4	21.6	0.988
Ag	eHWG-3	15.898	19.9	24.0	0.99121
Ag	eHWG-4	24.202	27.3	26.6	0.98554
AgI/Ag	eHWG-1	2.599	6.13	12.8	0.98636
AgI/Ag	eHWG-2	3.892	11.8	9.9	0.98832
AgI/Ag	eHWG-3	4.324	17.7	7.4	0.98423
AgI/Ag	eHWG-4	4.554	23.4	5.8	0.99146

**Table 3:** Summary of the performance of different IDs.

ID (mm)	AgI/Ag Waveguide	SD of Blank (dB*10^−3^)	Sensitivity (dB/ppm*10^−4^)	LOD (ppm)	Correlation Coefficient
0.7	eHWG-1	13.317	7.5	53.3	0.98066
0.7	eHWG-2	16.913	14	36.3	0.99145
0.7	eHWG-3	18.969	17	33.5	0.97047
0.7	eHWG-4	25.674	28.7	26.9	0.98524
1.4	eHWG-1	2.599	6.1	12.8	0.98636
1.4	eHWG-2	3.892	11.8	9.9	0.98832
1.4	eHWG-3	4.324	17.7	7.4	0.98423
1.4	eHWG-4	4.554	23.4	5.8	0.99146
2.0	eHWG-1	1.126	5.4	6.3	0.99643
2.0	eHWG-2	2.123	12.5	5.1	0.99595
2.0	eHWG-3	2.175	14.7	4.5	0.99499
2.0	eHWG-4	2.320	26	2.7	0.99702
